# Diffuse large B‑cell lymphoma followed by myelodysplastic syndrome with TP53 mutation: a case report and literature review

**DOI:** 10.3389/fonc.2026.1882599

**Published:** 2026-08-26

**Authors:** Xue Wu, Jingyuan Zhao, Yongli Fan, Xiaofeng Yang, Xinhong Yang

**Affiliations:** 1 Department of Hematology, the Affiliated Hospital of Chengde Medical College, Chengde, Hebei, China; 2 Hebei Key Laboratory of Panvascular Diseases, Chengde, China; 3 Department of Intensive Care Unit, the Affiliated Hospital of Chengde Medical College, Chengde, Hebei, China; 4 Department of Clinical Laboratory, the Chengde Hospital of Traditional Chinese Medicine, Chengde, Hebei, China; 5 Department of Pediatric Surgery, the Affiliated Hospital of Chengde Medical College, Chengde, Hebei, China

**Keywords:** allogeneic hematopoietic stem cell transplantation, autologous hematopoietic stem cell transplantation, diffuse large B-cell lymphoma (DLBCL), secondary myelodysplastic syndrome, therapy-related myeloid neoplasms, TP53 mutation

## Abstract

**Background:**

Secondary myelodysplastic syndrome (sMDS) is increasing as more individuals survive treatment for a primary cancer diagnosis, which is associated with many factors, such as prior alkylator therapy, topoisomerase II inhibitors and higher-dose pretransplant irradiation, hematopoietic cell transplantation (HCT) and graft purging.

**Materials and methods:**

We report a unique case of sMDS diagnosed 34 months after a sequential treatment regimen consisting of chemotherapy, autologous hematopoietic stem cell transplantation (auto-HSCT), and chimeric antigen receptor T (CAR-T) cell therapy for primary splenic diffuse large B-cell lymphoma (DLBCL). With reference to existing literature, we discuss plausible etiologic interpretations and research limitations.

**Results:**

The patient was diagnosed with therapy-related myelodysplastic syndrome (t-MDS) with multihit-TP53. The patient achieved complete remission after allogeneic hematopoietic stem cell transplantation (allo-HSCT) and achieved sustained complete remission with full donor chimerism during 18 months of follow-up.

**Conclusion:**

The case we reported highlights the cumulative risk of t-MDS associated with multi-modal intensive treatments for relapsed/refractory DLBCL, even with initial disease control. The etiology of sMDS is multifactorial. This case may provide novel hypothesis-generating clues for exploring the mechanisms underlying clonal evolution under multimodal hematopoietic stress. This single case raises the hypothesis that early allo-HSCT may contribute to favorable prognosis, which needs validation in larger cohorts.

## 1 Introduction

Diffuse large B-cell lymphoma (DLBCL) is an aggressive subtype of non-Hodgkin’s lymphoma (NHL), accounting for 30-40% of all lymphomas and the most common histological subtype globally (1). Rituximab plus CHOP (cyclophosphamide, doxorubicin, vincristine, and prednisolone) markedly improves survival, yet some patients experience relapse. For these patients, treatment modalities such as high-dose chemotherapy combined with autologous hematopoietic stem cell transplantation(auto-HSCT), chimeric antigen receptor T (CAR-T) cell therapy, and allogeneic hematopoietic stem cell transplantation (allo-HSCT) can be implemented. However, the incidence of secondary myeloid neoplasms (sMNs) after long-term treatment for NHL has been estimated at 1-1.5% per year from 2–10 years after the start of primary chemotherapy (2, 3). Herein, we report a unique case of TP53-mutated myelodysplastic syndrome (MDS) diagnosed 34 months after a sequential treatment regimen consisting of R-CHOP chemotherapy, auto-HSCT, and CD19 CAR-T cell therapy in a patient with primary splenic DLBCL. This report details the clinical course of this patient after multi-line intensive lymphoma treatment, contextualizes this rare case against published data, and underscores the value of timely allo-HSCT for such high-risk patients. We further explore potential pathogenesis and study limitations, especially the absence of baseline testing to confirm pre-existing TP53-mutant clonal hematopoiesis prior to treatment.

## 2 Case presentation

**Figure 1:**
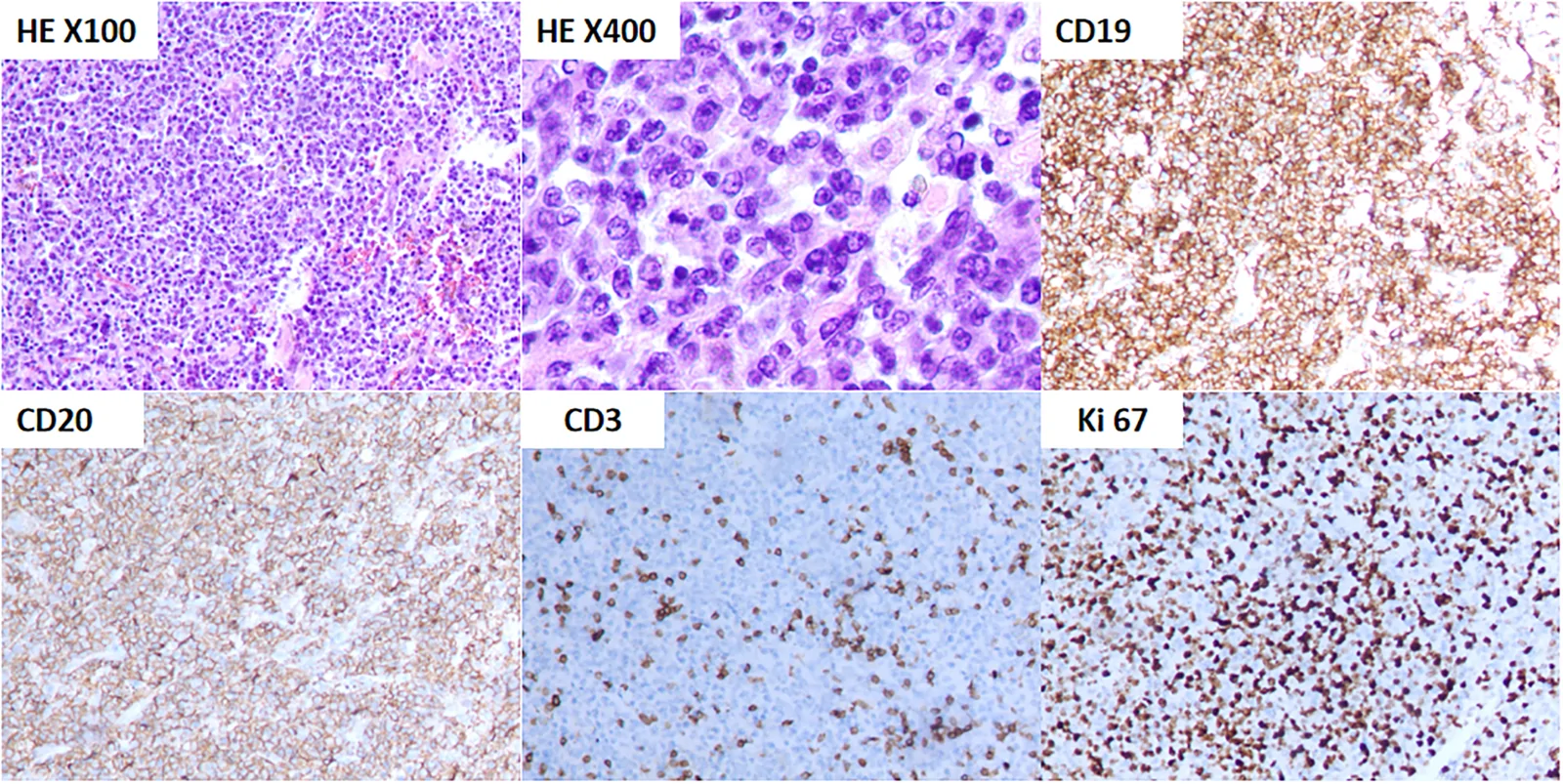
Hematoxylin and eosin (H&E) and immunohistochemistry staining of diffuse large B-cell lymphoma of the spleen.

On August 25, 2021, a 55-year-old male patient was admitted due to a 1-year history of splenic space-occupying lesion and a 7-day history of progressive enlargement of the mass. Peripheral blood laboratory test results were as follows: white blood cell (WBC) count 7.29×10^9^/L, hemoglobin (Hb) 93.00 g/L, platelet (PLT) count 171.00×10^9^/L, and lactate dehydrogenase (LDH) 550 U/L. Contrast-enhanced abdominal computerized tomography (CT) showed multiple lesions in the spleen and multiple nodules in the mesentery, which were suspicious for malignant lesions. Plain and enhanced CT of the neck demonstrated multiple enlarged cervical lymph nodes. Subsequent PET/CT revealed multiple FDG-hypermetabolic lesions suggestive of malignancy, including bilateral pulmonary opacities, splenomegaly with hypodense splenic parenchymal/hilar lesions, enlarged mesenteric lymph nodes, and bilateral pleural thickening. Patchy intestinal FDG uptake warranted close monitoring to exclude lymphoma infiltration. To confirm the pathological diagnosis, the patient underwent splenectomy. The immunohistochemical staining results of the splenic specimen were as follows: CD20 (+), CD19 (+), CD79a (+), Ki-67 (70% positive), Cyclin D1 (−), CD10 (−), Bcl-6 (partially positive), MUM1 (+), c-Myc (10% positive), Bcl-2 (+), CD30 (<10% positive), CD15 (−), CD21 (+), CD23 (positive in residual follicular dendritic cells), CD4 (−), CD8 (−), and EBV (−) (**Figure 1**). Final pathology confirmed non-germinal center splenic DLBCL. Bone marrow smear showed normal trilineage hematopoiesis, and karyotyping yielded a normal karyotype, 46, XY [15]. Combined with clinical and imaging findings, the lymphoma was Ann Arbor stage IVA with an International Prognostic Index (IPI) score of 3 (high-intermediate risk).

From September 2, 2021, he received four cycles of R-CHOP regimen chemotherapy. The specific doses were cyclophosphamide 750 mg/m², doxorubicin 50 mg/m², vincristine 1.4 mg/m² (maximum 2 mg), rituximab 375 mg/m² on day 1, and oral prednisolone 100 mg on days 1–5. And he achieved a complete remission (CR) observed on PET-CT. Given extensive multiorgan extranodal involvement and high-intermediate risk, standard six-cycle R-CHOP was deemed inadequate, so an individualized regimen was implemented. The patient received four cycles of R-CHOP induction and attained complete metabolic remission (CMR), followed by one cycle of R-EPOCH consolidation: rituximab 375 mg/m² IV day 1; 96-hour continuous infusion of etoposide 50 mg/m²/day, doxorubicin 10 mg/m²/day and vincristine 0.4 mg/m²/day days 1–4; oral prednisone 60 mg/m²/day days 1–5; cyclophosphamide 750 mg/m² IV day 5. Autologous stem cell collection was completed on December 24, 2021, followed by an extra R-CHOP consolidation cycle. Despite CMR, widespread extranodal lesions implied high risk of PET-CT-undetectable minimal residual disease. Upfront auto-HSCT was therefore delivered as intensified consolidation to lower long-term lymphoma relapse risk. The patient received standard full-dose BEAM conditioning beginning February 15, 2022, in preparation for auto-HSCT. The conditioning regimen comprised intravenous carmustine 300 mg/m² on day −8; intravenous etoposide 200 mg/m² once daily and cytarabine 200 mg/m² every 12 hours on days −7 to −4; and continuous pump infusion of melphalan 140 mg/m² on day −2. Intensive prophylactic hydration was performed on day −1 to reduce the risk of renal toxicity. Autologous peripheral blood stem cell reinfusion (transplant day 0) was conducted on February 23, 2022. The patient sustained continuous CR for almost 5 months post-transplantation, during which only a single dose of rituximab was administered for maintenance therapy.

On July 27, 2022, the patient was admitted again due to left axillary lymph node enlargement. Lymph node biopsy combined with immunohistochemistry was consistent with DLBCL, indicating relapse of the disease. Given the recurrent disease status, CAR-T cell therapy was scheduled, and patient samples were collected for pre-treatment correlative assessment. The patient was re-hospitalized on September 12, 2022, presenting with right testicular swelling. Leukapheresis was performed for autologous CAR-T cell manufacturing, and the patient received one cycle of CHOP regimen chemotherapy. On October 9, 2022, the patient received a lymphodepleting chemotherapy combination of fludarabine and cyclophosphamide. Three days later, autologous CD19-targeted CAR-T cells (total dose: 3.0×10^6^/kg; 99.87% CD3+ T cells and 57.07% CD3+ CAR-T cells) were infused. The administered CAR-T product, termed CAR19GG (batch number: CV017010201), was manufactured by Hebei Taihe Chunyu Biotechnology Co., Ltd., and remained an investigational product without commercial approval at the time of treatment. Post-infusion, peripheral CAR+ T cell levels gradually increased and peaked on day 13. Throughout the treatment and observation period, the patient did not develop cytokine release syndrome or immune effector cell-associated neurotoxicity syndrome. A PET-CT evaluation conducted on day 30 post CAR-T infusion confirmed CR. To reduce long-term lymphoma recurrence risk, oral zanubrutinib 160 mg twice daily was initiated on day 18 after CAR-T cell infusion for scheduled one-year maintenance therapy. The patient adhered to regular outpatient follow-up during the entire maintenance period, and serial systemic disease assessments continuously confirmed sustained lymphoma CR. However, persistent mild anemia was noted, with hemoglobin levels fluctuating between 100 g/L and 120 g/L.

On November 14, 2023, routine outpatient blood tests showed a further decrease in hemoglobin to 99 g/L. Bone marrow cytology presented no cellular morphological abnormalities, and no mitotic figures were observed on karyotyping; molecular genetic testing was not performed during this follow-up assessment. On March 12, 2024, the patient returned with worsening fatigue. Peripheral blood routine examination revealed WBC 7.6×10^9^/L, Hb 89 g/L, and PLT 232×10^9^/L. Repeat bone marrow aspiration showed unremarkable marrow morphology. No evaluable metaphases were obtained for karyotype analysis, and molecular genetic testing was again not performed.

**Figure 2:**
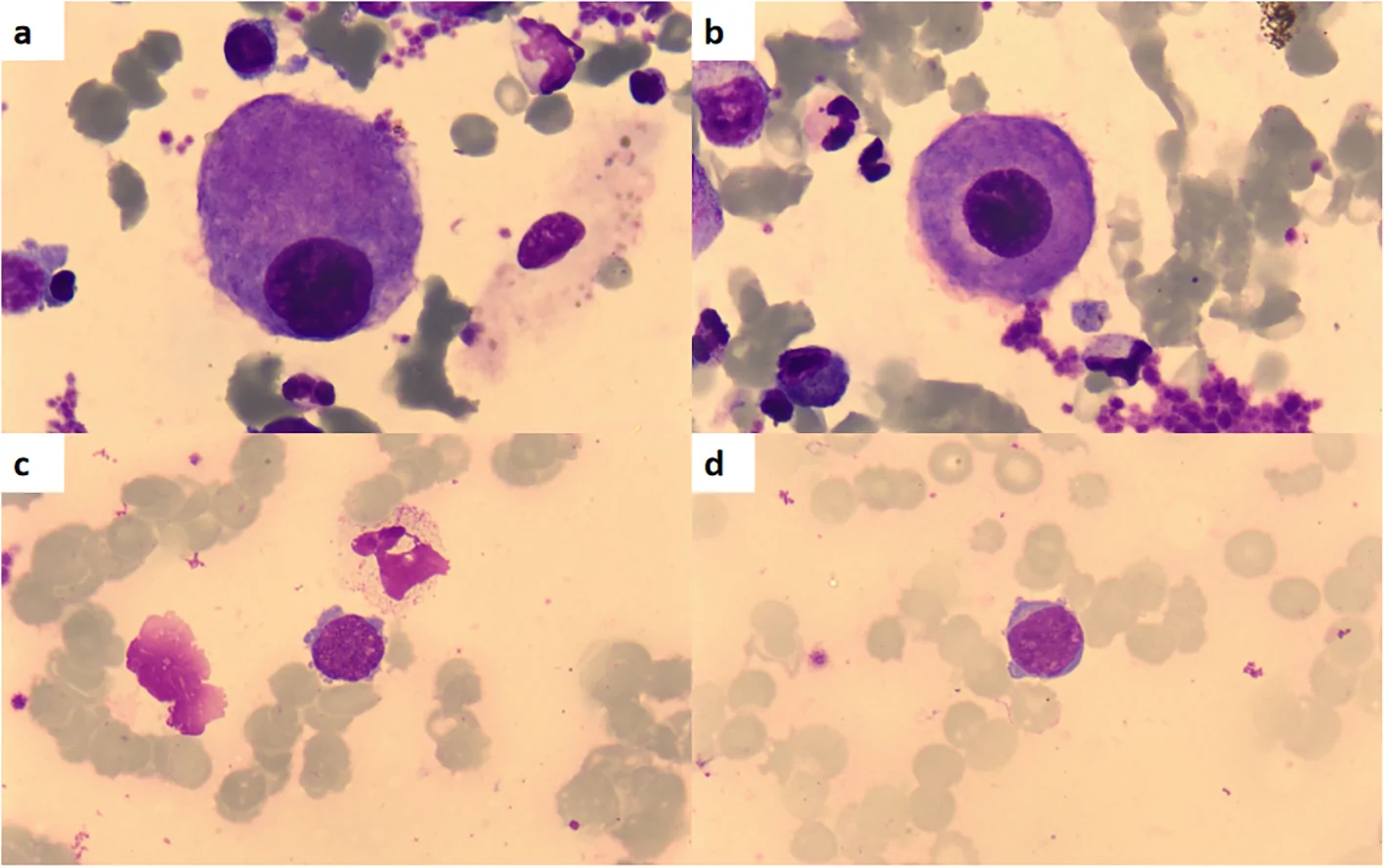
Bone marrow smears obtained from this patient upon diagnosis of MDS. **(A, B)** Bone marrow smears show mononuclear round megakaryocytes. **(C,D)** Bone marrow smear shows increased myeloblasts. (Wright–Giemsa stain, 100× objective).

**Figure 3:**
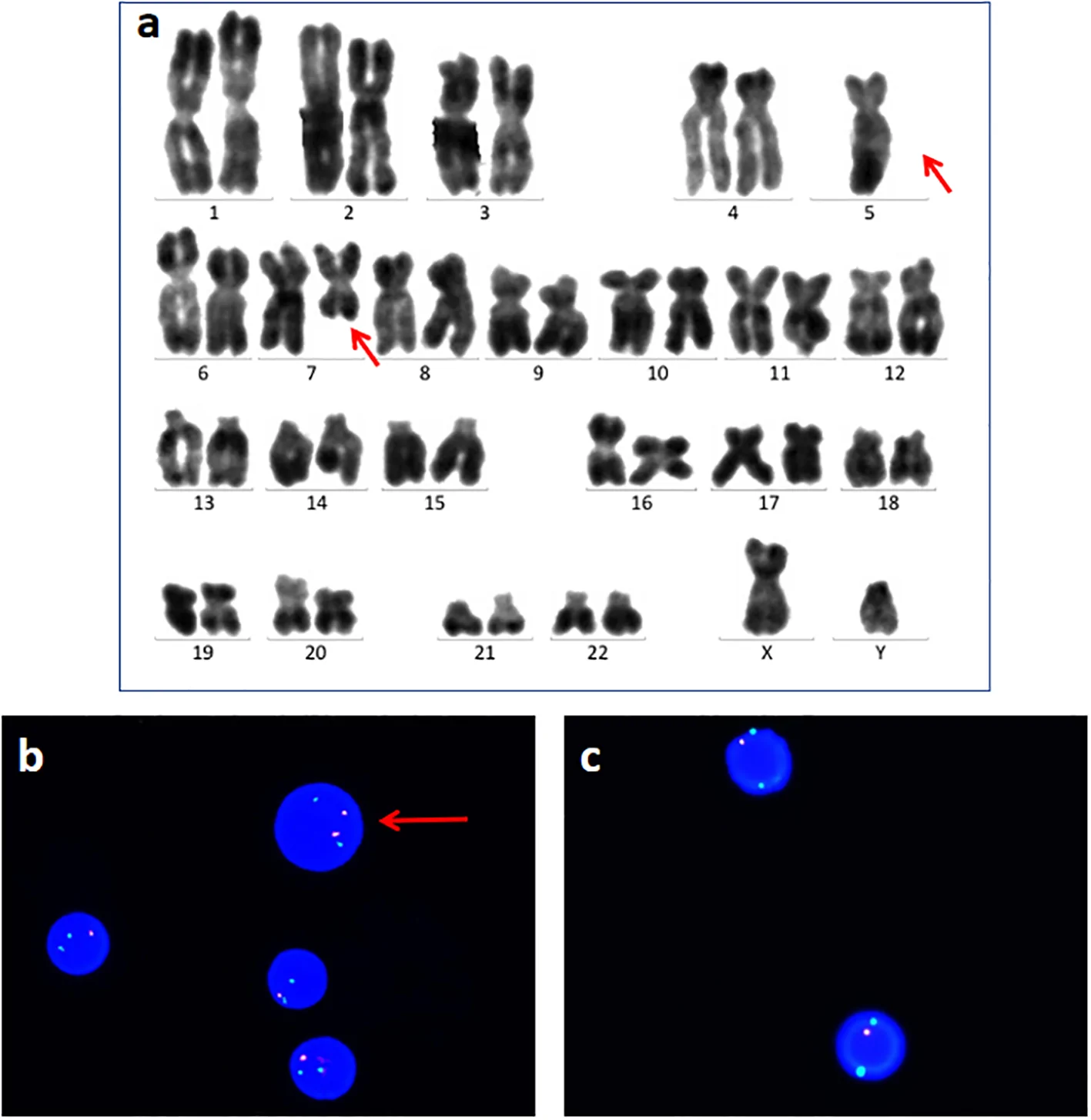
**(A)** A representative R-banded karyotype shows 45, XY, -5, del(7)(q22). **(B)** FISH demonstrates 5q deletion (5q-). Green probe targets 5p15, red probe targets 5q31. Positive cells display 1 red and 2 green signals (1R2G). The red arrow points to a negative cell (2R2G). **(C)** FISH demonstrates 7q deletion (7q-). Green probe targets the centromere of chromosome 7 (CEN7), red probe targets 7q31. Positive cells display 1 red and 2 green signals (1R2G).

On June 14, 2024, the patient was hospitalized with persistent and aggravated fatigue. Routine peripheral blood tests showed: WBC 9.8×10^9^/L, RBC 1.67×10¹²/L, Hb 48.0 g/L, and PLT 277×10^9^/L. Bone marrow smear examination identified dysmegakaryopoiesis, primarily characterized by increased mononuclear megakaryocytes (**Figure 2**). Flow cytometric (FCM) immunophenotyping detected no aberrant lymphoid or myeloid blast populations, and no immunophenotypic dysplastic features were observed in the granulocytic, erythroid, or megakaryocytic lineages. Chromosomal karyotype analysis revealed a karyotype of 45, XY, -5, del(7)(q22) [15]. Fluorescence *in situ* hybridization (FISH) further confirmed 7q deletion (7q-) and 5q deletion (5q-) aberrations (**Figure 3**). Targeted next-generation sequencing (NGS) using our in-house custom myeloid malignancy gene panel was performed, with an assay lower limit of detection (LLOD) at a variant allele frequency (VAF) of 3%. This sequencing assay identified a TP53 pathogenic variant on transcript NM_000546.6: c.373A>C, p.Thr125Pro (p.T125P), with a VAF of 91%. Based on the 2022 International Consensus Classification (ICC) of Myeloid and Lymphoid Neoplasms, the patient was definitively diagnosed with therapy-related myelodysplastic syndrome (t-MDS) with multihit-TP53. The International Prognostic Scoring System -Molecular (IPSS-M) score was >1.5, placing the patient in the very high risk category. After one cycle of oral lenalidomide treatment, the patient developed fever, decreased urine output and elevated serum creatinine, which was considered lenalidomide intolerance. Therefore, the treatment was switched to subcutaneous luspatercept 75 mg on July 11, 2024. Subsequently, the patient presented with recurrent fever and worsening fatigue and was hospitalized for further evaluation and management. Laboratory tests showed a WBC of 0.2×10^9^/L, Hb of 62 g/L, and PLT of 17×10^9^/L. BM examination revealed 12% myeloblasts (**Figure 2**), and FCM identified abnormal myeloblasts accounting for 10.82%, confirming disease progression. The patient was transferred to another hospital and received allogeneic hematopoietic stem cell transplantation (allo-HSCT) on September 21, 2024, with a 5/10 HLA-matched donor (his daughter). Regrettably, detailed information including the conditioning regimen, GVHD prophylaxis strategy, hematopoietic engraftment parameters and full post-transplant clinical course is therefore inaccessible.

**Figure 4:**
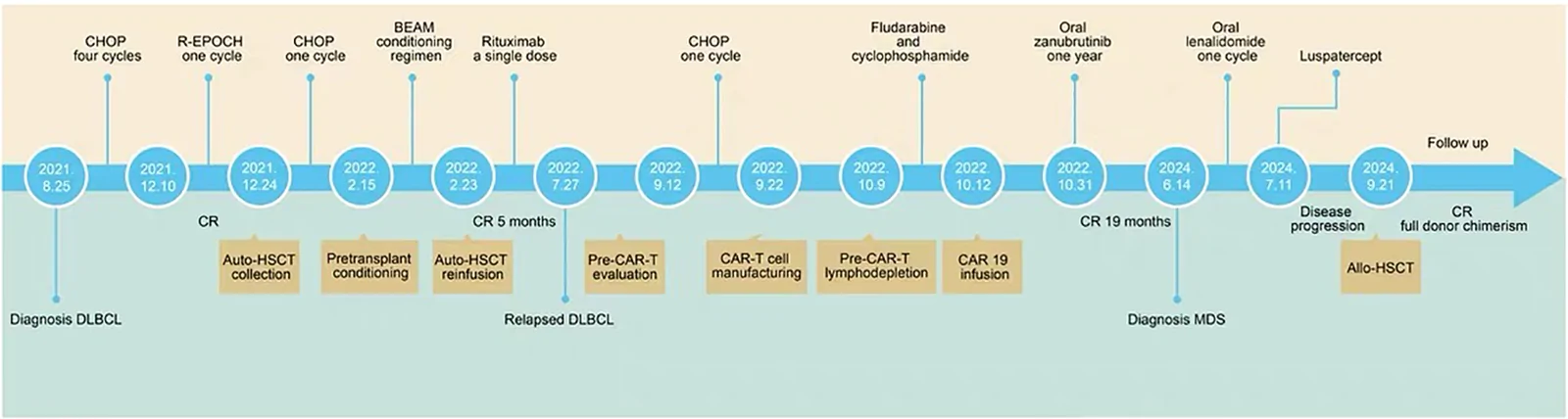
Timeline of key clinical events for the patient.

The patient returned to our hospital for the first follow-up on December 12, 2024. Clinical evaluation confirmed CR, and flow cytometry-based minimal residual disease (MRD) detection yielded negative results. Next-generation sequencing (NGS) showed no TP53 gene mutations. Short tandem repeat (STR) chimerism analysis confirmed full donor chimerism, with no recipient-derived cells or third-party allogeneic cells detected. At the time of manuscript submission, the patient maintained persistent CR and full donor chimerism, with a remission duration of more than 18 months. (**Figure 4**).

## 3 Discussion

Although the development of chemotherapy has prolonged the life expectancy of cancer patients, secondary hematological malignancies are a serious problem. The development of secondary hematological malignancies is associated with many factors. Therapy-related myeloid neoplasms (t-MN) are a well-recognized and heterogeneous group of clonal disorders occurring after chemotherapy and/or radiotherapy, encompassing t-MDS and therapy-related acute myeloid leukemia (t-AML). Depending on the chemotherapeutic agent and/or radiation, two main types of t-MN have been described (4). The first and most common subtype, occurring after exposure to alkylating agents and/or radiation with a latency period of 5–10 years, is frequently accompanied by unbalanced cytogenetic abnormalities, such as loss of all or parts of chromosomes 5 and/or 7. The second less common subtype, arising after treatment with topoisomerase II, has shorter latency period of 1–5 years and frequently exhibits balanced chromosomal rearrangements involving 11q23/MLL, 21q22/RUNX1, and PML-RARA. Factors such as age <35 years, prior alkylator therapy, topoisomerase II inhibitors and higher doses of pre-transplant irradiation, HSCT and graft purging have been associated with higher incidence of secondary myelodysplastic syndrome (sMDS) (5–8).

Auto-HSCT improves progression-free and overall survival for patients with lymphoproliferative and plasma cell disorders, yet intensive regimens raise secondary malignancy risk. Vaxman I et al. found that baseline disease status may interact with the risk of secondary malignancies following auto-HSCT. Patients with lymphoproliferative diseases (LPD) who received auto-HSCT had a higher risk of both overall malignancies and MDS/AML (9). The estimated risk of sMDS following auto-HSCT is 3.3 times higher than non-transplant (9, 10), and the EBMT reported a 5% incidence of secondary MDS/AML at 5 years post auto-HSCT in patients with LPD (11). Established risk factors include total body irradiation conditioning, advanced age, pre-transplant chemo-radiotherapy, ex vivo stem cell purging, and peripheral blood stem cell use instead of bone marrow (5, 12). Nevertheless, significant heterogeneity in pre-transplant characteristics and varied conditioning strategies hinder precise individualized risk evaluation for second malignancies, and also restrict further exploration of its underlying mechanisms.

Late adverse events of CAR-T therapy mainly include hypogammaglobulinemia, infections and second primary malignancies (SPMs) (13). Hematologic malignancies, especially sMNs, dominate post-CAR-T SPMs, followed by non-melanoma skin cancers. A pharmacovigilance study using FAERS (6,370 patients) and VigiBase (6,942 patients) confirmed MDS as the most prevalent SPMs, ahead of AML and T-cell lymphoma (14). Transient post-CAR-T cytopenia stems from inflammatory stress and lymphodepletion-induced myelosuppression, whereas persistent cytopenia strongly suggests emerging secondary myeloid malignancies (15). Nevertheless, the causal link and underlying mechanisms between CAR-T therapy and SPMs remain unclear. Prior DNA-damaging chemo/radiotherapy and hematopoietic stem cell transplantation further raise risks of high-risk karyotypic MDS/AML (15). It remains unclarified whether CAR-T products or therapy-induced immunosuppressive niches drive malignant clonal evolution. A MD Anderson cohort of 114 relapsed/refractory large B-cell lymphoma patients receiving CD19 CAR-T showed higher cumulative t-MN incidence in patients with clonal hematopoiesis (CH) (19%) versus those without CH (4.2%) (16). CAR-T is reserved for patients failing prior lines of therapy, whose elevated SPM risk is commonly attributed to preceding chemo- or stem cell transplantation (17). However, several studies propose that CAR-T genetic engineering plus lymphodepleting agents (fludarabine, cyclophosphamide, bendamustine) may predispose genetically vulnerable patients to novel secondary malignancies (18).

Chromosomal abnormalities involving chromosomes 5 and 7, which are characteristic of t-MN, often develop after exposure to alkylating agents, and are also quite common in patients who develop t-MN post-ASCT. It has been hypothesized that the G-C-rich regions of these chromosomes are specifically targeted for methylation of O6 guanines, which results in DNA breaks and subsequent deletions or truncations (19). Discrepancies between karyotyping showing monosomy 5 (−5) and FISH results indicating 5q− represent a typical pseudo-monosomy phenomenon involving chromosome 5 aberrations in myeloid neoplasms. Conventional R/G-banded cytogenetic analysis relies solely on chromosome enumeration and gross morphological features for karyotype interpretation, which carries limited resolution and fails to detect subtle cryptic chromosomal rearrangements and segmental translocations. The apparent monosomy 5 (−5) identified by R-banding in this case does not reflect complete loss of the entire chromosome 5. Instead, malignant cells harbor deletion of the critical 5q31 region; meanwhile, short-arm fragments of chromosome 5 are integrated into other chromosomes via unbalanced translocations to form derivative chromosomes (20). This reduces the number of intact chromosome 5 copies visible under microscopy, leading to the false classification of monosomy 5. In contrast, FISH utilizes locus-specific probes targeting 5p15.2 and 5q31 to precisely distinguish short-arm and long-arm abnormalities. Only deletion of the 5q31 locus is detected, yielding a 5q− pattern without evidence of full chromosome 5 loss. Galván et al. (21) reported that 85.7% of MDS/AML patients with karyotypic −5 carry only isolated 5q31 segmental deletion and genuine full-length chromosome 5 loss is extremely rare. This finding validates the limitations of banded karyotyping for identifying true monosomy 5 and highlights that FISH can correct cytogenetic classification biases to clarify the nature of underlying chromosomal aberrations.

It is now well established that TP53 mutations are early leukemogenic events. TP53 mutations have been observed to be present with a significantly higher frequency in t-MN than in *de novo* myeloid neoplasms and are hypothesized to play a critical role in disease pathogenesis (22). Studies have shown that chemotherapy does not directly induce TP53 mutations. In a subset of cases, TP53-mutant clones that would later progress to malignancy were detected even before the start of any cytotoxic therapy (23). This indicates that TP53-mutant clones preexist prior to treatment and acquire a proliferative advantage under the selective pressure of subsequent therapy. Among various treatments, exposure to auto-HSCT and chemotherapy was associated with a 2.2- and 2-fold higher risk of TP53 mutations t-MN, respectively, compared with radiation exposure alone (24). Studies have shown that the same TP53 mutation clone was detected in a subset of post-CAR-T t-MN cases, suggesting that CAR-T therapy may lead to rapid expansion of pre-existing clones through previously unexplained mechanisms (25). Regrettably, the patient in this case did not undergo gene mutation testing at the time of initial diagnosis of DLBCL, so it cannot be confirmed whether the patient had underlying clonal hematopoiesis prior to treatment. Therefore, whether the above therapies induce mutations or cause the expansion of the pre-existing mutation is unclear. Further research is needed to isolate the impact of these therapies on TP53-mutated myeloid neoplasms development and the mechanisms there of. Mutations in TP53, detected in over 20% of DLBCLs, are associated with poor prognosis and therapeutic resistance (26). Routine TP53 testing is recommended for newly diagnosed DLBCL. Further research should clarify how multimodal therapies trigger TP53-mutated myeloid neoplasms and their mechanisms, supporting etiologic study, risk stratification and treatment optimization.

In addition, the TP53 VAF reached 91% in pre-transplant bone marrow samples, while the result of this mutation was negative after allo-HSCT. These results in this patient imply that the genetic alteration predominantly affected hematopoietic cells and is of probable somatic origin. We proposed performing genetic analysis on non-neoplastic specimens such as nail clippings to exclude germline mutation, yet the patient declined our recommendation. Since no normal non-hematopoietic tissues were available for testing, the possibility of a germline TP53 mutation cannot be entirely eliminated. Two TP53 mutational states have been described based on the degree of TP53 allelic loss. In MDS, TP53 single-hit denotes a single TP53 mutation, whereas TP53 multi-hit is defined as ≥2 TP53 mutations, or one TP53 mutation accompanied by del(17p), monosomy 17, VAF ≥ 50%, or complex karyotype (27, 28). In addition, TP53 VAF has also been reported to be of prognostic significance in MDS. This is probably explained by the strong correlation between high VAF and biallelic targeting (29). Recent studies have reported that TP53 patients have poor responses to lenalidomide (30) and hematopoietic stem cell transplantation (HSCT) (31, 32), as well as marked but transient responses to hypomethylating agent (HMA) (33). The patient presented chiefly with anemia. While awaiting allo-HSCT, oral lenalidomide was empirically given for anemia based on the patient’s preference and local insurance policies. Though standard for isolated del(5q) MDS, lenalidomide has minimal efficacy in t-MDS with complex karyotypes or TP53 mutations, and the patient soon suffered rapid disease progression.

The etiology of myeloid neoplasms following B cell-directed immunotherapy is multifactorial, and its mechanisms remain incompletely elucidated. The patient reported herein initially received cyclophosphamide-, doxorubicin- and etoposide-based cytotoxic chemotherapy, followed by auto-HSCT and salvage CD19 CAR-T therapy for relapse. As described in previous studies, the altered bone marrow microenvironment induced by cytotoxic therapies exerts selective pressure, leading to the expansion of pre-existing hematopoietic clones (34). Such a complex pathogenetic process and sequential therapy regimen is rarely documented in the literature. The etiology of secondary MDS is multifactorial. Therefore, it remains challenging to clarify which specific therapeutic intervention contributed to the subsequent onset of MDS. Collectively, this case may provide novel hypothesis-generating clues for exploring the mechanisms underlying clonal evolution under multimodal hematopoietic stress.

The treatment of patients with t-MN presents some challenges unique from those seen in *de novo* myeloid malignancies. Currently, there is no standardized treatment regimen for t-MN. For t-MN patients with favorable cytogenetic abnormalities (inv (16), t (16;16), t (8;21)), standard AML induction chemotherapy combined with high-dose cytarabine consolidation is recommended (35). Allo-HSCT is recognized as the only curative approach for the majority of t-MN patients. For those deemed to be transplant candidates, initial treatment with standard induction chemotherapy can be attempted and may be expected to achieve a similar rate of CRs as *de novo* cases with the same karyotypes (4). Those achieving a CR should proceed directly to transplant if a donor is available as further cycles of chemotherapy only raise the significant risk of additional morbidity and mortality in this population with limited reserve (36). However, the feasibility of allo-HSCT is restricted by damage to hematopoietic stem cells from prior radiotherapy and chemotherapy, cumulative organ toxicity of chemotherapeutic agents, and limitations in donor selection. Even after undergoing allo-HSCT, the overall outcome for patients with t-MN remains poor. It has been reported that with current treatment approaches the median overall survival for these patients continues to be within the range of only 6–9.7 months (4). Allo-HSCT is indicated as soon as possible for patients with higher-risk MDS. In the present case, given disease progression and an elevated proportion of myeloid blasts, the patient underwent timely allo-HSCT, which significantly improved the patient’s prognosis. During the 18-month follow-up after transplantation, the patient remained in CR with sustained full donor chimerism.

## 4 Conclusion

In conclusion, this case reveals the cumulative myelotoxic risk of layered intensive anti-lymphoma therapies, even when lymphoma achieves sustained complete remission. Insidious progressive isolated anemia was an easily missed early warning of t-MDS, and the high TP53 variant allele frequency highlights its core pathogenic role in t-MN. Limitations include the absence of baseline clonal hematopoiesis testing at DLBCL onset, precluding confirmation of pre-existing mutant clones or attribution of t-MDS to one single treatment modality.

We propose four actionable recommendations for clinicians treating high-risk DLBCL patients receiving multi-line intensive therapy:1. Screen myeloid genes and clonal hematopoiesis at initial DLBCL diagnosis to stratify long-term t-MN risk; 2. Maintain long-term blood count surveillance post auto-HSCT/CAR-T and perform bone marrow evaluation for persistent unexplained anemia; 3. Rapidly search for donors and assess allo-HSCT eligibility upon diagnosis of high-risk multi-hit TP53 t-MDS; timely allo-HSCT is the only curative option; 4. Inform patients of cumulative t-MN risks before administering multiple intensive therapies.

This case provides translational clues to investigate clonal hematopoietic evolution under serial bone marrow stress and informs standardized long-term monitoring and therapeutic decision-making for high-risk DLBCL survivors.

## Data Availability

All datasets generated and/or analysed during the current study are not publicly available due to patient‑privacy restrictions but are available from the corresponding author upon reasonable request.
